# Socioeconomic inequality in awareness, treatment and control of diabetes among adults in India: Evidence from National Family Health Survey of India (NFHS), 2019–2021

**DOI:** 10.1038/s41598-023-29978-y

**Published:** 2023-02-20

**Authors:** Suraj Maiti, Shamrin Akhtar, Ashish Kumar Upadhyay, Sanjay K. Mohanty

**Affiliations:** International Institute for Population Sciences, Mumbai, India

**Keywords:** Socioeconomic scenarios, Epidemiology, Diabetes complications, Type 2 diabetes

## Abstract

Diabetes is a growing epidemic and a major threat to most of the households in India. Yet, there is little evidence on the extent of awareness, treatment, and control (ATC) among adults in the country. In this study, we estimate the prevalence and ATC of diabetes among adults across various sociodemographic groups and states of India. We used data on 2,078,315 individuals aged 15 years and over from the recent fifth round, the most recent one, of the National Family Health Survey (NFHS-5), 2019–2021, that was carried out across all the states of India. Diabetic individuals were identified as those who had random blood glucose above 140 mg/dL or were taking diabetes medication or has doctor-diagnosed diabetes. Diabetic individuals who reported diagnosis were labelled as aware, those who reported taking medication for controlling blood glucose levels were labelled as treated and those whose blood glucose levels were < 140 mg/dL were labelled as controlled. The estimates of prevalence of diabetes, and ATC were age-sex adjusted and disaggregated by household wealth quintile, education, age, sex, urban–rural residence, caste, religion, marital status, household size, and state. Concentration index was used to quantify socioeconomic inequalities and multivariable logistic regression was used to estimate the adjusted differences in those outcomes. We estimated diabetes prevalence to be 16.1% (15.9–16.1%). Among those with diabetes, 27.5% (27.1–27.9%) were aware, 21.5% (21.1–21.7%) were taking treatment and 7% (6.8–7.1%) had their diabetes under control. Across the states of India, the adjusted rates of awareness varied from 14.4% (12.1–16.8%) to 54.4% (40.3–68.4%), of treatment from 9.3% (7.5–11.1%) to 41.2% (39.9–42.6%), and of control from 2.7% (1.6–3.7%) to 11.9% (9.7–14.0%). The age-sex adjusted rates were lower (*p* < 0.001) among the poorer and less educated individuals as well as among males, residents of rural areas, and those from the socially backward groups Among individuals with diabetes, the richest fifth were respectively 12.4 percentage points (pp) (11.3–13.4; *p* < 0.001), 10.5 pp (9.7–11.4; *p* < 0.001), and 2.3 pp (1.6–3.0; *p* < 0.001) more likely to be aware, getting treated, and having diabetes under control, than the poorest fifth. The concentration indices of ATC were 0.089 (0.085–0.092), 0.083 (0.079–0.085) and 0.017 (0.015–0.018) respectively. Overall, the ATC of diabetes is low in India. It is especially low the poorer and the less educated individuals. Targeted interventions and management can reduce the diabetes burden in India.

## Introduction

Diabetes, a non-reversible chronic condition, is now a common disease. It is the major cause of mortality and morbidity, leading to increased treatment costs across the globe^1–3^. In 2021 alone, over 6.7 million deaths were attributed to diabetes, globally^4^. The International Diabetes Federation (IDF) estimates that 537 million people worldwide were living with diabetes in 2021; this number is projected to increase to 643 million by 2030 if no effective preventive measures are adopted ^4^. Over 541 million people are at an elevated risk of getting diabetes. During 1990–2016, there was a more than two-fold increase in Disability Adjusted Life Years (DALYs) related to diabetes^5^. While the prevalence of diabetes has increased rapidly in almost all countries, people from low-and middle-income countries (LMCs) alone account for 75% of the diabetics worldwide^4,6,7^.

The prevalence, growth and distribution of diabetes vary largely across countries. Diabetes is associated with almost every chronic disease. It complicates medical treatment and aggravates chronic conditions. Over time, diabetes can cause serious heart conditions and damage to eyes, kidneys, and nerves, increasing the risk of limb amputation, loss of vision, and early death^8^. The global health expenditure on diabetes was estimated at USD 966 billion in 2021 and is projected to increase to USD 1028 billion by 2030^4^. Target 3.4 of the Sustainable Development Goals (SDGs) adopted by the United Nations (UN) is to reduce premature mortality owing to non-communicable diseases (NCDs) by one-third, which cannot be achieved without the prevention and control of diabetes^9–11^. In 2021, the World Health Organization (WHO) launched the Global Diabetes Compact, a global initiative aimed at sustained improvements in diabetes prevention and care, with a special focus on people living in LMCs^12,13^.

Early detection can reduce the burden of diabetes and can be the key to better quality of life. Given the non-reversible nature of the disease, increasing awareness, treatment, and control (ATC) is key to reduce its burden. ATC has a strong socioeconomic gradient. As per Hart’s inverse care law, individuals with the highest need are least likely to receive healthcare^14,15^. In general, individuals with a low socioeconomic status (SES) less aware, less treated, and in less control of diabetes. A growing number of studies globally have analysed country-specific prevention, awareness, treatment and control of diabetes and found low ATC among individuals living in low socio-economic conditions^16–18^. During 2011–2012, 80.6% of South African adults with diabetes had an unmet need for care^19^. In Bangladesh, in a study on adults 35 years and over, it was found that among individuals with diabetes, 41.2% were aware, 36.9% were being treated, and 14.2% had controlled diabetes^20^. A cross-sectional survey was conducted in Northeast China in 2012 among individuals in the age bracket of 18 to 79 years. It found the ATC rates of diabetes to be 64.1%, 52.9% and 44.2% respectively^21^. In a study conducted in a semi-urban area of Nepal among adults aged 25 years and over in 2016–2017, the ATC rates of diabetes were found to be 65%, 94% and 21% respectively^22^. A recent report shows a consistent rise in the prevalence of diabetes across Latin America with 50% awareness^23^.

India is home to the world's second highest number of diabetic patients. Within the age group of 20–79 years, India has 74.9 million diabetics in 2021 projected to increase to 124.9 million by 2045^4^. According to IDF, one out of every seven diabetic adults worldwide resides in India, and one in every third household has diabetic patients^4^. In the case of India, there have been very few nationally-representative studies for diabetes^24–33^. A population-based study of 1.3 million adults, carried out during 2012–2014, estimated a 7.5% diabetes prevalence in India^34^. Indian Council of Medical Research-India Diabetes (ICMR-INDIAB) is a population-based cross-sectional study, carried out during 2008–2015 in three phases in 15 states of India observed that the prevalence of diabetes varied widely between the states and was higher in the low SES groups in the urban areas of developed states^30,35,36^. The prevalence of diabetes in adults aged 20 years and above in India increased from 5.5% in 1990 to 7.7% in 2016^25^. According to a much recent report by the National NCD Monitoring Survey (NNMS), the diabetes prevalence in India stood at 9.3% in 2018^24^. Similar estimates have been given by IDF, where diabetes prevalence was estimated at 9.6% in 2021 and projected to increase to 10.4% by 2030^4^.

In this study, we estimate the prevalence, awareness, treatment, and control of diabetes at the national and state levels using the nationally-representative National Family Health Survey (NFHS-5) conducted in 2019–2021^37^. We also examine socioeconomic inequalities that arise in evaluating diabetic care.

## Methods

### Data source

We used data from the fifth round of the National Family Health Survey (NFHS-5), a nationally-representative household-based survey conducted during 2019–21 in India. The survey was done across 707 districts in 28 states and 8 union territories of India. A total of 2,843,917 individuals from 636,699 households were successfully interviewed. Among the 2,843,917 individuals, 2,078,315 were adults above 15 years age.

NFHS-5 used a stratified two-stage sampling method. In the first stage, within each district, the sampling process was carried out differently in rural and urban areas. In rural areas, villages were used as primary sampling units (PSUs), which were selected using probability proportional to size (PPS), whereas in urban areas, census enumeration blocks (CEBs), selected with PPS systematic sampling, were used as PSUs. In the second stage, in every selected rural and urban cluster, 22 households were randomly selected with systematic random sampling after the complete mapping and household listing of the selected PSUs. The detailed methodology followed by NFHS-5 can be found in the NFHS India Report^37^.

### Measures

All adults aged 15 years and above were requested to undergo a finger-stick blood glucose measurement using the Accu-Chek Performa glucometer with glucose test strips for random blood glucose testing by trained health investigators. An individual was classified as having high blood glucose if they had a random blood glucose level of 141–160 mg/dL and as having very high blood glucose if they had a random blood glucose level of more than 160 mg/dL. For our purpose, we combined the high blood glucose and very high blood glucose categories, and defined the combined category as having a high blood glucose level. An individual was classified as having a diagnosis of diabetes if they had responded with “yes” to the question “*Told high blood glucose on two or more occasions by doctor or health professionals?*”. An individual was classified as taking medication for diabetes if they had responded with “yes” to the question “*Currently taking any prescribed medicine to lower blood glucose?*”.

For our analysis, an individual was ascertained as having diabetes if they had high blood glucose (above 140 mg/dL) or if they were diagnosed with diabetes or if they took some medication for lowering their blood glucose. We classified individuals with diabetes as (a) “Aware” if they reported having been diagnosed with diabetes; (b) “Treated” if they reported taking medication for lowering blood glucose; and (c) “Controlled” if they were taking medication for lowering blood glucose and had their random blood glucose levels were under the threshold for diabetes used in this study (< 140 mg/dL). The blood glucose thresholds used to ascertain diabetes was determined as per the NFHS-5 India Report^37^.

We used various socio-demographic attributes as independent variables in this analysis. The household wealth index was used as the primary indicator of socioeconomic status and was constructed based on the principal component analysis (PCA). It used a set of variables, including housing characteristics, household amenities and household ownership of durable goods. The wealth quintiles were calculated by assigning a score to each usual (de jure) household member, ranking each person in the household population according to their score and dividing the distribution into five equal categories, each with 20% of the population^37^.

Additionally, the study included socio-demographic variables ascertaining an individual’s age (15–29, 30–44, 45–59, 60–74, and 75 + years), sex (male, female), household size (1–3, 4–6, and 7 & above members), educational attainment (no education, primary, secondary, and higher), caste (Schedule Caste (SC), Schedule Tribe (ST), Other Backward Class (OBC), and Other), religion (Hindu, Muslim, Christian, and Other), place of residence (urban, rural), marital status (unmarried, currently married, and other), alcohol (drinking, not drinking) and tobacco (using, not using) consumption status, and state fixed effect. Our analytical sample covered 2,078,315 adults above 15 years age.

### Statistical Analysis

We used the full sample of individuals aged 15 years and over to estimate diabetes prevalence. A subsample of these participants who were identified as having diabetes was used to estimate the rates of ATC. We estimated the prevalence and the ATC rates of diabetes nationally, by state, by household wealth quintile group and by other socio-demographic characteristics. We adjusted the estimates for age and sex using the age-sex composition of the full sample as used in literature^38^. Descriptive statistics, along with bivariate analysis, were used to observe the distribution and association of the variables. F-statistic values along with a 95% confidence interval, were presented in the results.

We used concentration indices (covariance between an outcome and rank in distribution of household wealth) and concentration curves to quantify the wealth disparity among the individuals, using the full distribution of the household wealth score^39,40^. The concentration indices were adjusted for age and sex. Multivariable logistic regression was used to estimate the adjusted marginal effects of the various independent variables and state indicators on the probability of having diabetes and the probabilities of ATC among those with diabetes. Each marginal effect was averaged over the sample used in the respective regressions.

Utilizing the *svyset* package of Stata (version 16.0), sampling weights were applied throughout the analyses to account for stratification and cluster sampling to ensure that the findings were nationally representative. Confidence intervals were provided for each estimate at the 95% level. All the individuals with a complete response to blood glucose testing, diabetes diagnosis, treatment, wealth quintile, and all the other reported covariates were included in the study sample. State estimates of ATC were presented using maps generated in R version 4.1.1, using the *ggplot2* package^41^.

### Ethics statement

The International Institute for Population Sciences (IIPS), Mumbai, provided the ethical approval of NFHS-5 (2019–21). Additionally, the ICF International Review Board (IRB) looked over the survey and gave ethical approval. The respondents provided signed consent after being fully informed about the survey's purpose and procedures. Only interviews were done after obtaining proper consent from each participant. The Demographic and Health Surveys (DHS) Program's website hosts the NFHS-5, an anonymous dataset that is made available to the public and cannot be used to identify the survey respondents.

## Results

**Figure 1:**
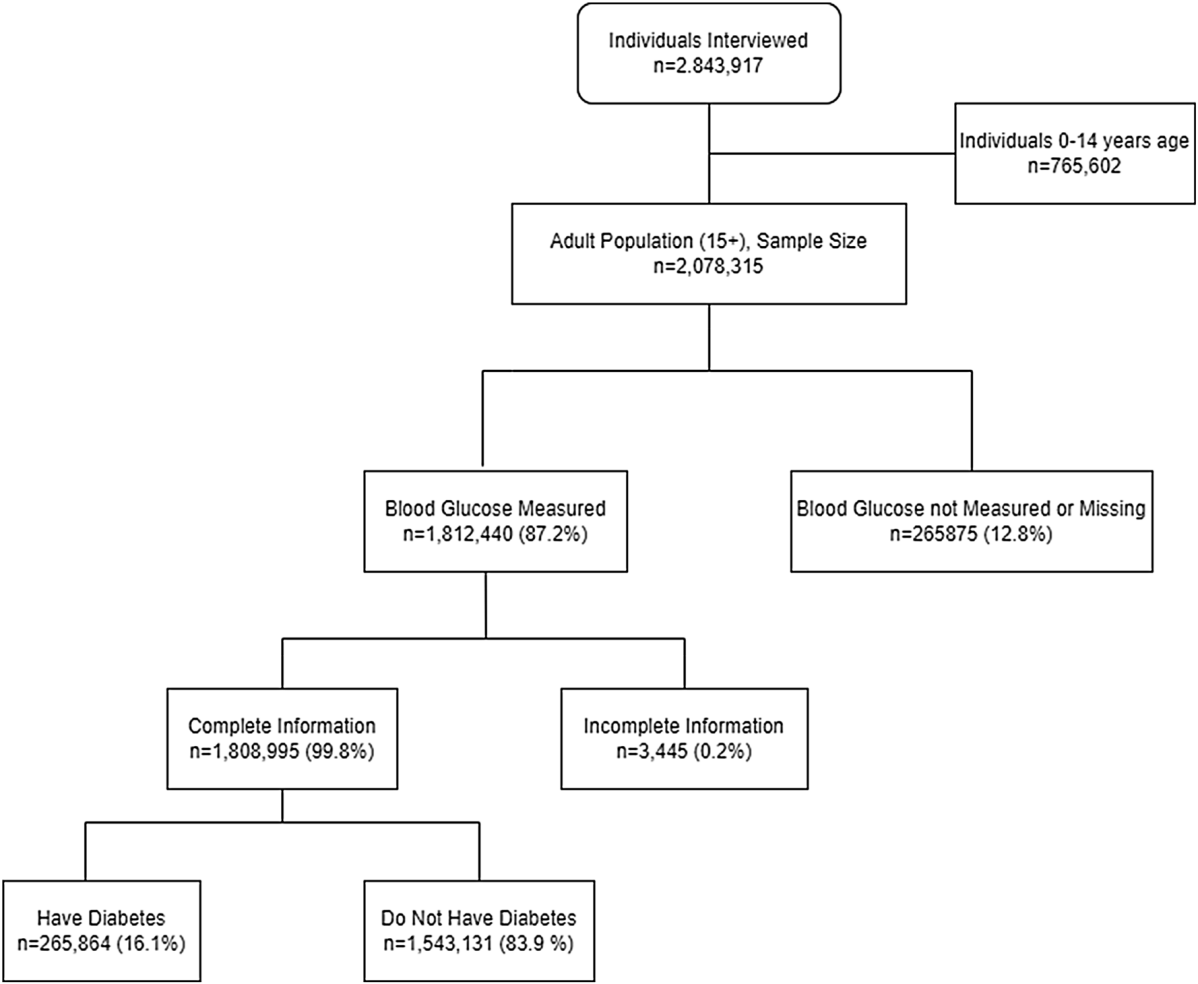
Flowchart of participant selection.

The selection of the analysis sample was done as shown in Fig. 1. Out of 2,078,315 adults, blood glucose was measured for 1,812,440 (87.20%) participants. The rest of the participants either did not give consent, or were interviewed by proxy, or had some physical limitations barring them from blood glucose measurement. Among those whose blood glucose was measured, 3,445 (0.2%) individuals did not report all the information on diagnosis or treatment or on the sociodemographic characteristics, leaving the analysis sample to have 1,808,995 participants with full response, which was then used to estimate diabetes prevalence. Within this sample, 265,864 (16.1%) individuals were identified as having diabetes and had their data used to estimate the rates of diabetes ATC.

Table 1 shows the characteristics of the full analysis sample and the estimates of the age-sex adjusted prevalence of diabetes. Women made up the majority of the responders (53.6%). Half of the sample had secondary level education. The sample was made up primarily of married (69.4%) and rural (68.4%) people. With an average household size of 4–6 people, more than half of the sample belonged to underprivileged classes.

**Table 1:** Participant characteristics and adjusted diabetes prevalence among adults in India, 2019–21.

	Participants		Diabetes prevalence		F-stat (p-value)
Characteristics	n	%	%	95% CI	
Overall	1,808,995	100.0	16.1	[15.9,16.1]	
Wealth Quintile					
Poorest	374,555	18.4	13.1	[12.9,13.3]	391.14(< 0.001)
Poorer	397,856	20.0	14.3	[14.1,14.5]	
Middle	377,699	20.7	15.8	[15.6,16.0]	
Richer	348,695	20.9	17.6	[17.3,17.8]	
Richest	310,190	20.0	18.8	[18.5,19.1]	
Education					
No Education	471,754	25.1	13.2	[13.0,13.2]	718.4 (< 0.001)
Primary	249,542	14.0	16.5	[16.2,16.6]	
Secondary	858,014	46.9	17.9	[17.7,18.1]	
Higher	229,685	14.1	18.2	[17.8,18.5]	
Age, years					
15–29	638,081	35.1	5.1	[5.0,5.2]	12,032.8 (< 0.001)
30–44	504,550	27.6	13.2	[13.0,13.4]	
45–59	387,121	21.3	25.2	[24.9,25.4]	
60–74	227,003	13.0	32.7	[32.3,33.0]	
75 +	52,240	2.9	33.3	[32.7,33.9]	
Sex					
Male	844,591	46.4	16.8	[16.6,16.9]	361.5 (< 0.001)
Female	964,404	53.6	15.4	[15.2,15.4]	
Location					
Rural	1,368,285	68.4	14.9	[14.7,15.0]	648.6 (< 0.001)
Urban	440,710	31.6	18.5	[18.2,18.7]	
Caste					
SC	347,548	21.7	15.2	[14.9,15.4]	318.0 (< 0.001)
ST	342,194	9.5	12.7	[12.4,12.9]	
OBC	674,072	42.0	16.2	[16.0,16.3]	
Other	445,181	26.8	17.6	[17.3,17.8]	
Religion					
Hindu	13,375,813	82.4	15.7	[15.6,15.8]	123.1 (< 0.001)
Muslim	209,977	12.2	17.7	[17.3,18.0]	
Christian	132,674	2.6	20.2	[19.6,20.8]	
Other	90,531	2.8	14.2	[13.7,14.6]	
Marital Status					
Unmarried	410,822	22.0	14.9	[14.5,15.2]	24.5 (< 0.001)
Currently Married	1,249,302	69.6	16.2	[16.0,16.3]	
Other	148,871	8.4	16.1	[15.8,16.3]	
Alcohol Usage					
Not Drinking	1,589,858	90.5	16.2	[16.0,16.3]	109.1 (< 0.001)
Drinking	219,137	9.5	14.9	[14.6,15.1]	
Tobacco Status					
Not Using	1,341,921	77.1	16.8	[16.6,16.9]	724.3 (< 0.001)
Using	467,074	22.9	14.2	[14.0,14.3]	
Household Size					
Less Than 3	397,921	22.5	17.4	[17.2,17.6]	162.8 (< 0.001)
4–6	1,165,031	63.5	15.7	[15.5,15.8]	
7 +	246,043	14.1	15.0	[14.7,15.1]	

We estimated the prevalence of diabetes among people aged 15 years and older to be 16.1% (15.9–16.1%). The prevalence increased with age and was higher for males (16.8% (16.6–16.9%)) than for females (15.4% (15.2–15.4%)). Adjusted for age and sex, the estimated diabetes prevalence increased significantly when moving from the poorest quintile (13.1% (12.9–13.3%)) to the richest quintile (18.8% (18.5–19.1%)). The prevalence increased from 13.2% (13.0–13.2%) for those without a formal education to 18.2% (17.8–18.5%) for those with the highest levels of education. People in urban areas had a greater prevalence of diabetes (18.5% (18.2–18.7%)) than in rural areas(14.9% (14.7–15.0%)). The prevalence was also higher among those who belonged to the "other" castes, those with small families (less than 3 members), and those who were married.

**Figure 2:**
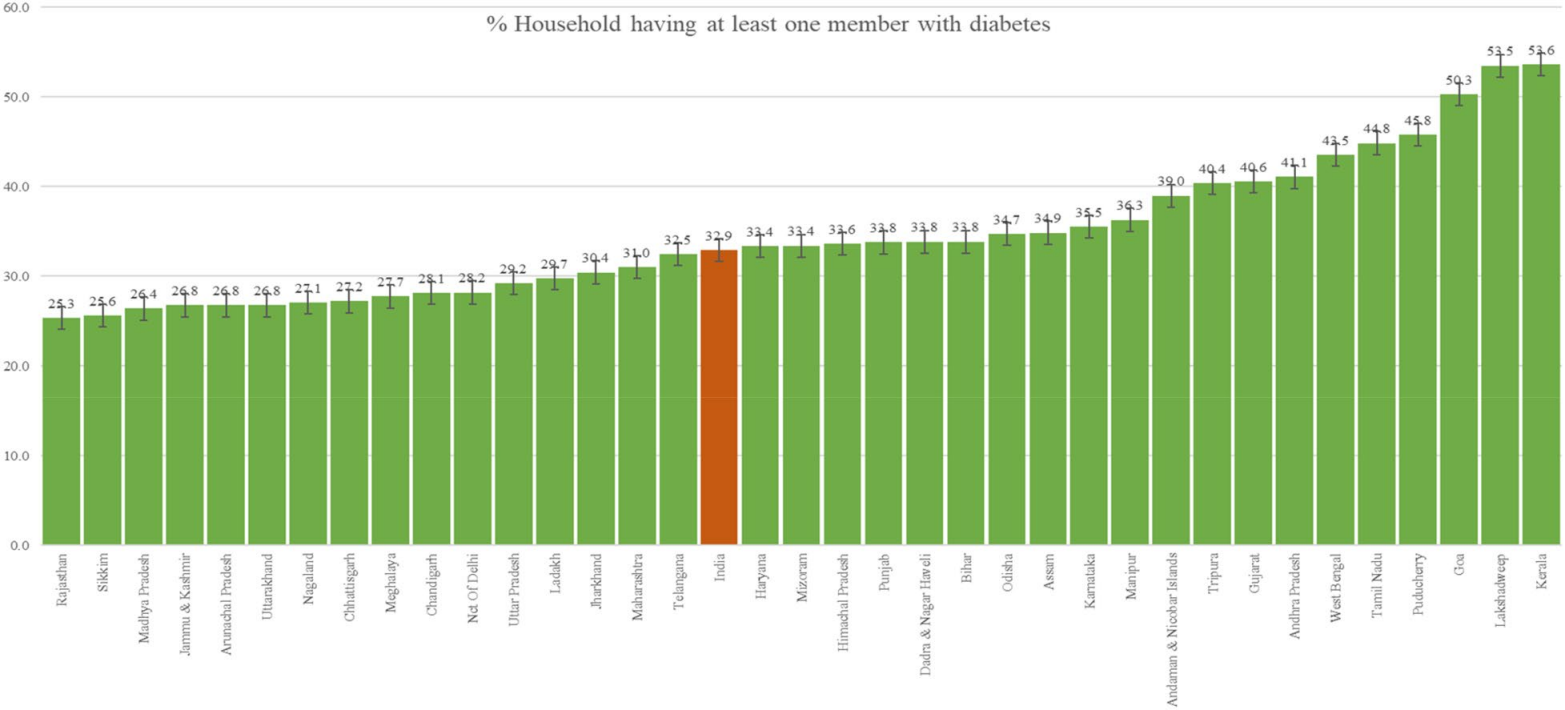
Proportion of households with at least one diabetic member across states of India, 2019–2021 (see Supplementary Table S1).

In Fig. 2, we show the proportion of households with at least one diabetic member across the states ofIndia. There exist stark observable differences across the households, with Rajasthan being the least affected state (25.3% (24.5–26.2%)) and Kerala the most affected (53.6% (52.6–54.6%)). On average in India, 32.9% (31.2–34.6%) of the households had at least one diabetic member, which is nearly one third of all households in India. Out of all the states of India, more than half of the states had diabetic households more than the national average. The economically prosperous state of Goa and the southern states of Andhra Pradesh, Tamil Nadu, and Kerela had more than 40% households with diabetic individuals.

Table 2 shows the adjusted percentage of diabetics aged 15 years and over who (a) were “aware” of this health condition; (b) were under “treatment” for it; and (c) had their blood glucose level under “control”. In total, 27.5% (27.1–27.9%) diabetics were found to be aware, 21.5% (21.1–21.7%) to be seeking treatment, and just 7% (6.8–7.1%) to be in control. The difference in awareness between the lowest and the highest wealth quintiles was 23 percentage points (pp). The rich-poor divide was the same for treatment and control, at 21 pp and 6 pp, respectively. Females were more likely than males to be aware of having diabetes, receiving treatment for it, and maintaining control over diabetes. ATC rates were also lower for STs, rural residents, unmarried people, and those with large families (more than 7 members). In comparison to being aware or receiving treatment, participants in younger age groups had more control.

**Table 2:** Adjusted percent aware, treated and controlled among those with diabetes among adults in India, 2019–21.

n = 265,864									
Characteristics	Awareness		F (p-value)	Treatment		F (p-value)	Control		F (p-value)
	%	95% CI		%	95% CI		%	95% CI	
Overall	27.5	[27.1,27.9]		21.5	[21.1,21.7]		7.0	[6.8,7.1]	
Wealth Quintile									
Poorest	14.1	[13.5,14.7]	660.8 (< 0.001)	9.9	[9.4,10.3]	897.8 (< 0.001)	4.7	[4.3,4.9]	97.0 (< 0.001)
Poorer	20.3	[19.6,20.9]		14.1	[13.6,14.5]		5.8	[5.4,6.0]	
Middle	26.3	[25.6,26.9]		19.8	[19.2,20.2]		6.7	[6.3,6.9]	
Richer	31.6	[30.9,32.3]		25.3	[24.6,25.8]		7.5	[7.2,7.5]	
Richest	37.3	[36.5,38.0]		30.8	[30.1,31.4]		9.1	[8.6,9.4]	
Education									
No Education	19.5	[19.0,19.9]	602.2 (< 0.001)	14.4	[14.0,14.7]	644.9 (< 0.001)	5.4	[5.1,5.6]	77.1 (< 0.001)
Primary	26.9	[26.2,27.5]		21.1	[20.5,21.6]		6.8	[6.4,7.0]	
Secondary	32.5	[31.9,32.9]		26.5	[26.0,26.9]		8.0	[7.7,8.3]	
Higher	37.7	[36.5,38.8]		29.4	[28.4,30.3]		8.7	[8.1,9.2]	
Age, years									
15–29	15.7	[14.9,16.4]	647.7 (< 0.001)	10.6	[10.0,11.1]	941.8 (< 0.001)	8.9	[8.3,9.4]	115.0 (< 0.001)
30–44	19.8	[19.1,20.3]		12.8	[12.4,13.2]		4.9	[4.6,5.1]	
45–59	29.5	[29.0,30.0]		23.6	[23.0,24.0]		6.4	[6.0,6.6]	
60–74	35.3	[34.7,35.9]		29.5	[28.9,30.0]		8.4	[8.0,8.6]	
75–89	33.5	[32.4,34.6]		27.6	[27.4,29.6]		8.9	[8.5,9.8]	
Sex									
Male	25.8	[25.3,26.2]	223.8 (< 0.001)	20.4	[20.0,20.7]	87.2 (< 0.001)	6.6	[6.3,6.7]	36.5 (< 0.001)
Female	29.3	[28.7,29.7]		22.5	[22.1,22.8]		7.4	[7.2,7.6]	
Location									
Rural	23.9	[23.4,24.3]	508.8 (< 0.001)	17.7	[17.4,18.0]	966.5 (< 0.001)	8.3	[7.9,8.5]	112.7 (< 0.001)
Urban	33.7	[32.9,34.4]		27.8	[27.2,28.3]		6.3	[6.1,6.4]	
Caste									
SC	25.4	[24.6,26.1]	231.9 (< 0.001)	18.9	[18.3,19.4]	239.4 (< 0.001)	6.6	[6.2,6.8]	21.2 (< 0.001)
ST	16.3	[15.3,17.1]		12.7	[11.9,13.3]		5.5	[5.0,5.9]	
OBC	29.3	[28.7,29.9]		22.5	[22.0,22.8]		7.1	[6.8,7.3]	
Other	28.9	[28.3,29.5]		23.6	[23.0,24.1]		7.5	[7.1,7.8]	
Religion									
Hindu	26.8	[26.3,27.2]	72.7 (< 0.001)	20.7	[20.3,21.0]	89.7 (< 0.001)	6.9	[6.6,7.0]	15.9 (< 0.001)
Muslim	28.3	[27.2,29.3]		23.1	[22.1,23.9]		7.1	[6.6,7.5]	
Christian	40.9	[39.0,42.8]		33.6	[31.9,35.1]		10.2	[9.2,11.1]	
Other	28.4	[26.9,29.8]		22.1	[20.9,23.3]		6.3	[5.5,7.0]	
Marital Status									
Unmarried	26.5	[25.0,27.9]	12.1 (< 0.001)	21.1	[19.7,22.4]	8.9 (< 0.001)	7.3	[6.5,8.0]	0.6 (0.5250)
Currently Married	27.9	[27.4,28.3]		21.8	[21.4,22.0]		7.0	[6.8,7.1]	
Others	26.2	[25.4,26.8]		20.3	[19.7,20.9]		6.8	[6.4,7.2]	
Alcohol Usage									
Not Drinking	27.9	[27.5,28.3]	70.5 (< 0.001)	21.9	[21.5,22.1]	102.9 (< 0.001)	7.1	[6.9,7.2]	11.9 (0.0005)
Drinking	24.4	[23.5,25.2]		18.1	[17.4,18.8]		6.3	[5.8,6.6]	
Tobacco Status									
Not Using	30.6	[30.1,31.0]	1279.3 (< 0.001)	24.2	[23.8,24.5]	1393.8 (< 0.001)	7.5	[7.3,7.7]	131.4 (< 0.001)
Using	19.8	[19.3,20.2]		14.6	[14.2,15.0]		5.6	[5.3,5.8]	
Household Size									
Less Than 3	29.3	[28.6,29.9]	37.9 (< 0.001)	23.4	[22.8,23.8]	82.2 (< 0.001)	7.3	[7.0,7.6]	
4–6	27.0	[26.5,27.4]		21.0	[20.6,21.3]		6.9	[6.7,7.1]	6.2 (0.002)
7 +	25.3	[24.5,26.1]		18.3	[17.6,18.9]		6.5	[6.1,6.9]	

**Figure 3:**
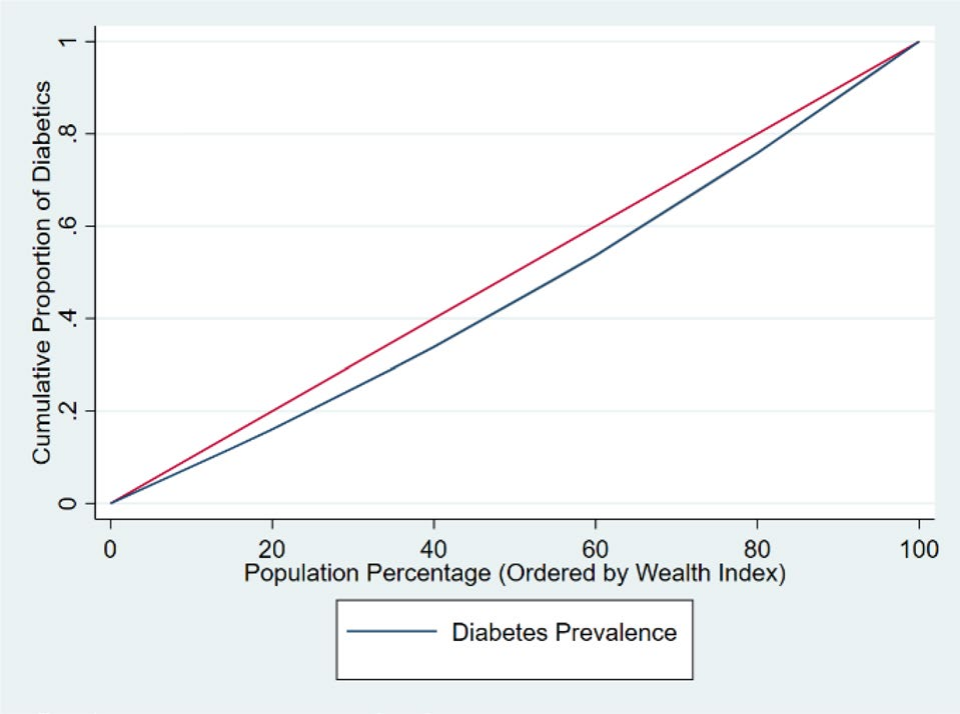
Adjusted concentration curve for diabetes prevalence in India, 2019–2021.

**Figure 4:**
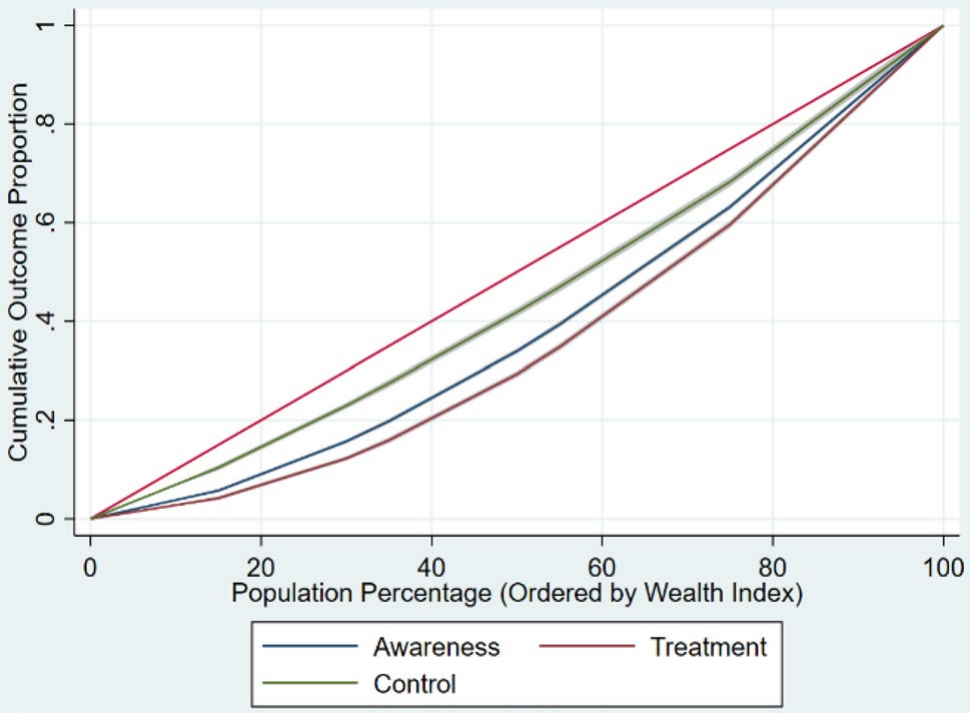
Adjusted concentration curves for ATC among those with diabetes in India, 2019–2021.

The age-sex adjusted concentration indices for diabetes and for ATC among diabetics aged 15 years and above are shown in Table 3. Diabetes prevalence concentration indices and ATC concentration indices were all positive, reflecting pro-rich inequality. The concentration curves for the prevalence of diabetes and for ATC among those with diabetes are shown in Figs. 3 and 4. We can deduce from Fig. 4 that the likelihood that an individual is aware of having diabetes, is getting treated, or has the disease under control decreases with increasing poverty.

**Table 3:** Adjusted concentration indices for diabetes and for ATC among those with diabetes in India, 2019–21.

Variables	Wagstaff concentration index (95% CI)	n
Diabetes Prevalence	0.023 [0.022,0.024]	1,808,995
Awareness	0.089[0.085,0.092]	265,864
Treatment	0.083 [0.079,0.085]	265,864
Control	0.017 [0.015,0.0183]	265,864

**Figure 5:**
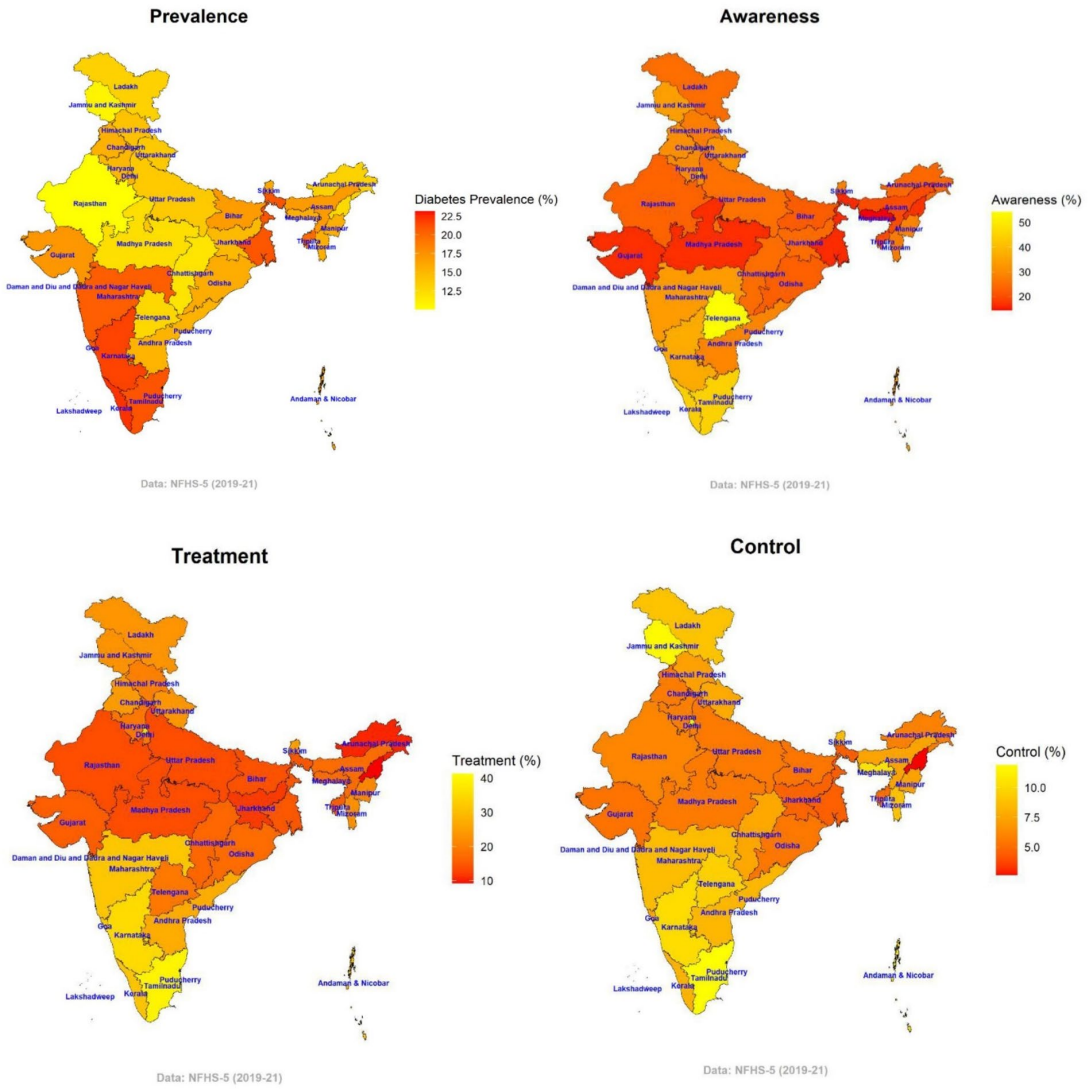
Adjusted diabetes a) prevalence and percent b) aware c) treatement and d) control among diabetic adults by states in India, 2019–2021 (see Supplementary Table S2).

The state-level variation in age-sex adjusted diabetes prevalence and rates of ATC among individuals with diabetes is shown in Fig. 5. In 15 of the 36 states, the prevalence of diabetes was higher than the 16.1% national average and ranged from 10.0% (9.6–10.4%) in Rajasthan to 23.2% (22.6–23.7%) in Lakshadweep. Diabetes awareness levels ranged from 14.4% (12.1–16.8%) in Meghalaya to 54.4% (40.3–68.4%) in Telangana. Treatment rates varied from 9.3% (7.5–11.1%) in Nagaland to 41.2% (39.9–42.6%) in Lakshadweep. The percentage of diabetics with controlled blood sugar varied from 2.7% (1.6–3.7%) in Nagaland to 11.9% (9.7–14.0%) in Tamil Nadu and was below the national average of 7% in 21 out of 36 states.

**Figure 6:**
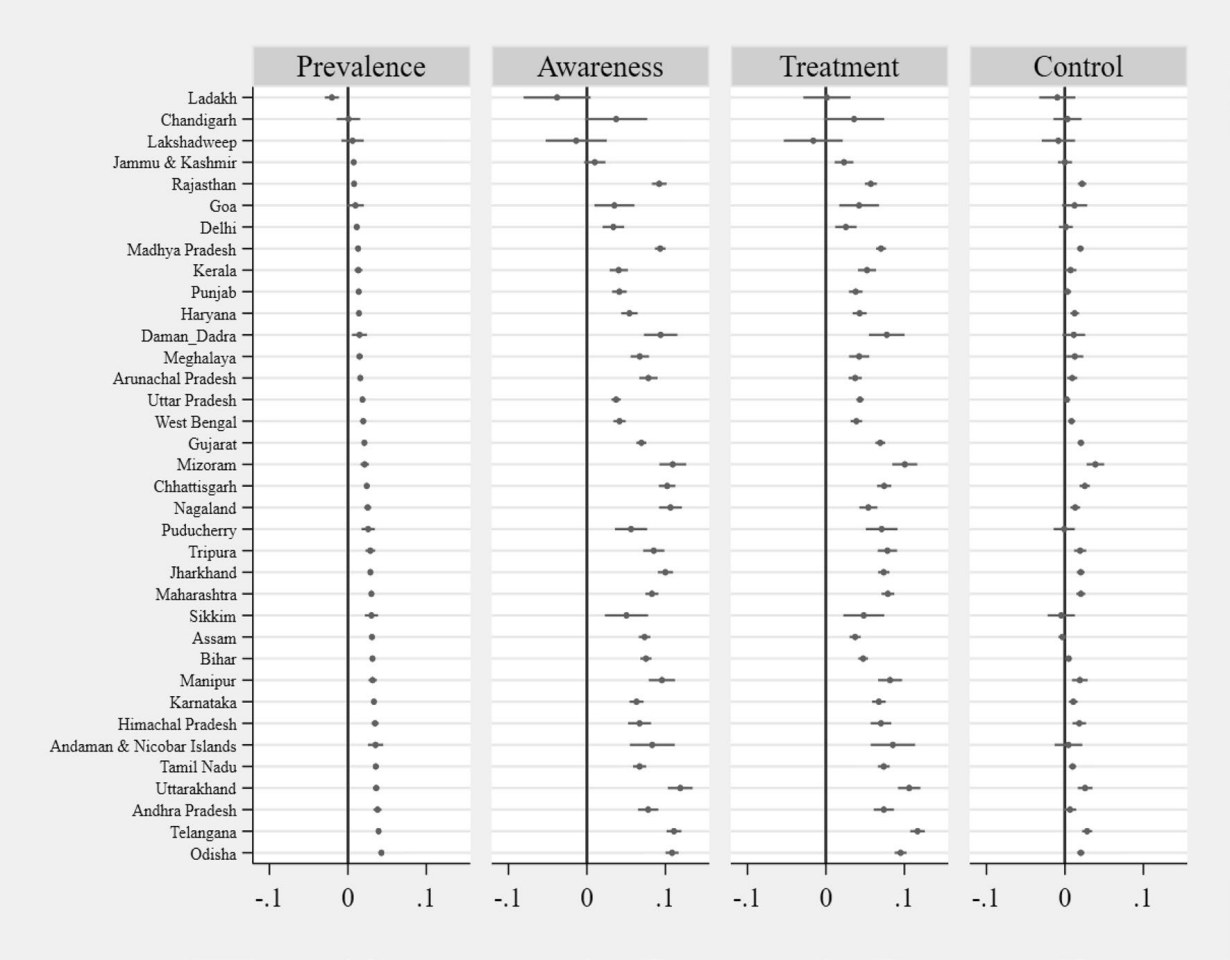
Adjusted concentration indices for diabetes and for ATC among persons who have the disease by states in India, 2019–2021 (see Supplementary Table S3).

Figure 6 displays the adjusted concentration indices for diabetes and for ATC among persons who have the disease by state, ranked from lowest to highest. With the exception of one state, this index's point estimate is positive, pointing a proportionately greater prevalence of diabetes among those with higher incomes. The majority of the 95% confidence bands do not contain 0, which is consistent with inequality. Similar results were found for ATC, with the exception of a small number of states, showing that those who were better off were more likely to be aware of having diabetes, seek treatment for it, and have it under control in those states.

**Figure 7:**
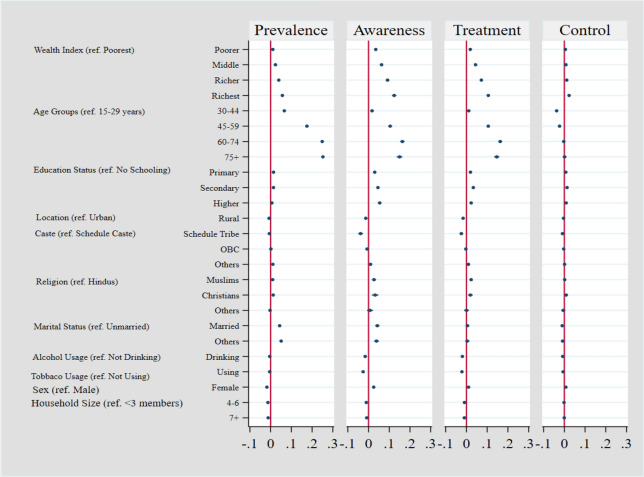
Averaged marginal effects on probability of diabetes and on probabilities of ATC among those with diabetes (see Supplementary Table S4).

For each of the outcomes, multivariable logistic regressions average marginal effects are shown in Fig. 7. After adjusting for sociodemographic traits and states, it was determined that people in the richest fifth had a 5.7 pp (5.3–6.1) greater prevalence of diabetes than those in the lowest fifth. A greater prevalence was seen in elderly persons, people who lived in urban areas, those with tiny nuclear families, and married people. Socioeconomic differences in ATC remained unchanged even after controlling for features and state. According to estimates, the ATC among the poorest and the richest fifths differed by 12.4 pp (11.3–13.4), 10.5 pp (9.7–11.4) and 2.3 pp (1.6–3.0) respectively. After controlling for other factors, the outcomes remained better for women, nuclear families, and urban dwellers. While keeping all other variables constant, awareness and treatment were higher among older persons when compared to control among the same older individuals.

## Discussion

We estimated the prevalence of diabetes among individuals aged 15 years and older at 16.1% (15.9–16.1%) based on the nationally-representative sample of NFHS-5. Among the individuals with diabetes, it was observed that there exists significant differences in prevalence, awareness, treatment and control of diabetes in the population. In all, 27.5% (27.1–27.9%) adults in India were aware of, 21.5% (21.1–21.7%) sought treatment, and just 7% (6.8–7.1%) had control over their diabetes^28,32,34,42^. As per the NNMS study, the prevalence of diabetes was at 9.3% among adults^24^ and as per a study based on NFHS-4 data, the prevalence only 3% for the age group 15–49 years^32^. The novelty of our study lies in the fact that we used the full sample of individuals above 15 years of age interviewed in NFHS-5, 2019–2021. This large sample of individuals gave us robust estimates for the prevalence and ATC of diabetes. The fact that our estimates are much higher than those of other small-scale studies^25,28,29,32,34,42,43^ may be attributed to the increasing prevalence of diabetes and the use of different methods used in estimation.

We found significant disparities in diabetes prevalence, awareness, treatment, and control across geographic and socioeconomic groups. High-income states like Maharashtra, Telengana, and Andhra Pradesh as well as states in the advanced stages of the demographic transition like Kerala, had the highest prevalence rates. Some of the poorer states had a relatively lower prevalence of diabetes. The extent of undiagnosed, untreated and uncontrolled diabetes is likely to be higher in the poorer states of India.

The SES inequality in the ATC of diabetes is high. Only 14.1% of the poorest fifth diabetics were aware of their condition, compared to 37.3% of the richest fifth and only 9.9% of the former received treatment compared to nearly 30.8% of the latter. The concentration of ATC is pro-rich. Therefore, the likelihood of diagnosis was lower for the poor.

Not only did people who were poorer and less educated have lower rates of ATC of diabetes, but so did those who were young (not the control group), male, living in rural areas, and single and had larger families. In this study, we predicted a higher prevalence of diabetes, as well as higher rates of ATC. Age is an incremental factor for awareness and treatment, but not for control. Therefore, there is an urgent need to implement interventions that prevent diabetes, aimed at early detection, and making use of newly devised treatments to delay progression to serious complications. Our findings that men and socioeconomically disadvantaged populations have a lower incidence of ATC are in line with earlier studies^32^.

India's adult population has low awareness of diabetes, which highlights the need for better health monitoring and education. Diabetes treatment and control rates are low and suboptimal^35^, especially among the poor and in the rural regions, which may be due to the barriers to healthcare access and the high cost of treatment. There are low-cost glycemic medications available, but the poorest individuals cannot even afford them. Health is a state subject in India, which explains the apparent state-level variances in ATC. The primary stakeholder in funding, operating facilities, providing manpower, and supplying pharmaceuticals is the state government. The central government can only make rules and offer support for some health-related services. Increased funding for diabetes management from the central government and the state governments is urgently needed. Some studies have previously pointed out the need for preparedness in the primary and secondary health care centres in India, for tracking non-communicable diseases^43^. The state of healthcare remains in utter despair, resulting in the rural residents being severely handicapped and marred with challenges compared to their urban counterparts^35,43^. The National Programme For Prevention And Control Of Cancer, Diabetes, Cardiovascular Diseases And Stroke (NPCDCS), is a programme set up by Government of India (GoI) to control NCDs including diabetes, which has been fruitful so far but has some shortcomings too^44^. The programme has been able to meet some of its targets of early detection and screening of NCDs like diabetes, which is key to resolving complications at an initial stage^45^. As pointed out by the recent ICMR-INDIAB study, across India, there is suboptimality in the achievement of targets related to tackling diabetic conditions, and a very low proportion of individuals are able to achieve all three of ATCs^35^. Monitoring at the population level can be achieved using the care cascade method of representing the number of people that pass through each stage of diagnosis, treatment, and control^32^. ATC across the states can be increased by educating the masses and by placing checks for early detection and monitoring.

The primary limitation of the study is that the diagnosis of diabetes was made based on a single finger stick random blood glucose measurement and may differ from the gold standard of HbA1c testing. Secondly, the fact that there was a very small proportion of non-respondents or people whose blood glucose sample could not be collected, however, this potential bias cannot offset our estimates.

Despite these limitations, this paper provides comprehensive estimates on the prevalence, awareness, treatment, and control of diabetes in India. These findings may be helpful in monitoring and designing national guidelines for the control and management of diabetes in the country.

## Supplementary Information

Supplementary Information.

## Data Availability

The data is freely available from https://dhsprogram.com/data/dataset/India_Standard-DHS_2020.cfm?flag=0.
