# Supplementary material for: Socioeconomic inequality in awareness, treatment and control of diabetes among adults in India: Evidence from National Family Health Survey of India (NFHS), 2019–2021
Source: Sci Rep. 2023 Feb 20;13:2971. doi: 10.1038/s41598-023-29978-y (PMC9941485; doi:10.1038/s41598-023-29978-y)
Supplement: Supplementary file 1 — Supplementary Information. [file 41598_2023_29978_MOESM1_ESM.docx]

**Supplementary File**

**Supplementary Table S1: Proportion of households with at least one diabetic in states of India, 2019-21**

| **State** | **% (95 % CI)** |
| --- | --- |
| Andaman & Nicobar Islands | 39.0 [42.4,35.5] |
| Andhra Pradesh | 41.1 [42.2,40.0] |
| Arunachal Pradesh | 26.8 [28.0,25.5] |
| Assam | 34.9 [35.7,34.0] |
| Bihar | 33.8 [34.6,33.1] |
| Chandigarh | 28.1 [31.3,25.0] |
| Chhattisgarh | 27.2 [28.2,26.2] |
| Daman and Diu and Dadra and Nagar Haveli | 33.8 [37.7,30.0] |
| Goa | 50.3 [54.8,45.7] |
| Gujarat | 40.6 [44.0,37.1] |
| Haryana | 33.4 [34.2,32.5] |
| Himachal Pradesh | 33.6 [34.6,32.7] |
| Jammu & Kashmir | 26.8 [28.5,25.0] |
| Jharkhand | 30.4 [31.7,29.2] |
| Karnataka | 35.5 [36.5,34.5] |
| Kerala | 53.6 [54.6,52.6] |
| Ladakh | 29.7 [30.8,28.7] |
| Lakshadweep | 53.5 [58.6,48.3] |
| Madhya Pradesh | 26.4 [27.1,25.7] |
| Maharashtra | 31.0 [31.9,30.1] |
| Manipur | 36.3 [37.8,34.7] |
| Meghalaya | 27.7 [29.4,26.0] |
| Mizoram | 33.4 [35.4,31.4] |
| Nagaland | 27.1 [28.6,25.5] |
| Nct Of Delhi | 28.2 [29.5,26.8] |
| Odisha | 34.7 [35.5,33.9] |
| Puducherry | 45.8 [49.2,42.4] |
| Punjab | 33.8 [34.8,32.8] |
| Rajasthan | 25.3 [26.2,24.5] |
| Sikkim | 25.6 [28.5,22.7] |
| Tamil Nadu | 44.8 [46.0,43.7] |
| Telangana | 32.5 [34.1,30.8] |
| Tripura | 40.4 [40.9,39.9] |
| Uttar Pradesh | 29.2 [30.4,28.0] |
| Uttarakhand | 26.8 [27.9,25.6] |
| West Bengal | 43.5 [44.2,42.8] |
| **India** | 32.9 [34.6,31.2] |

**Supplementary Table S2: Adjusted Diabetes prevalence and ATC among those with diabetes, across states of India, 2019-21**

| **States** | **Diabetes Prevalence** | **95% CI** | **Awareness** | **95% CI** | **Treatment** | **95% CI** | **Control** | **95% CI** |
| --- | --- | --- | --- | --- | --- | --- | --- | --- |
| Jammu & Kashmir | 10.7 | [10.1,11.3] | 34.1 | [31.3,36.9] | 23.2 | [20.7,25.6] | 11.6 | [9.7,13.5] |
| Himachal Pradesh | 14.3 | [13.5,15.0] | 27.9 | [25.6,30.2] | 20.3 | [18.6,22.0] | 7.3 | [6.3,8.3] |
| Punjab | 15.5 | [15.0,16.0] | 31.0 | [29.4,32.7] | 24.5 | [23.2,25.8] | 5.7 | [5.0,6.3] |
| Chandigarh | 19.8 | [17.3,22.3] | 34.2 | [28.1,40.4] | 29.8 | [24.4,35.2] | 4.7 | [1.8,7.6] |
| Uttarakhand | 13.7 | [13.1,14.3] | 31.9 | [29.8,34.1] | 23.8 | [21.7,25.9] | 7.7 | [6.3,9.1] |
| Haryana | 15.0 | [14.5,15.5] | 30.3 | [28.2,32.4] | 19.0 | [17.9,20.1] | 5.8 | [5.2,6.5] |
| Delhi | 15.6 | [14.9,16.2] | 41.6 | [39.2,43.9] | 37.4 | [35.2,39.5] | 11.4 | [10.1,12.7] |
| Rajasthan | 10.0 | [9.6,10.4] | 23.2 | [21.2,25.2] | 13.7 | [12.7,14.7] | 6.0 | [5.4,6.7] |
| Uttar Pradesh | 13.7 | [13.5,14.0] | 25.3 | [24.0,26.5] | 13.7 | [13.1,14.4] | 5.6 | [5.2,5.9] |
| Bihar | 16.9 | [16.5,17.3] | 22.3 | [21.0,23.6] | 14.3 | [13.4,15.1] | 5.4 | [4.9,5.9] |
| Sikkim | 16.1 | [14.4,17.8] | 28.6 | [25.2,31.9] | 24.8 | [21.4,28.3] | 8.6 | [6.2,11.0] |
| Arunachal Pradesh | 13.2 | [12.5,13.9] | 24.0 | [20.8,27.2] | 10.6 | [9.2,11.9] | 5.9 | [4.8,6.9] |
| Nagaland | 12.6 | [11.8,13.4] | 17.2 | [14.1,20.3] | 9.3 | [7.5,11.1] | 2.7 | [1.6,3.7] |
| Manipur | 15.6 | [14.8,16.4] | 28.7 | [26.6,30.8] | 21.3 | [19.3,23.4] | 7.3 | [6.2,8.5] |
| Mizoram | 15.6 | [14.6,16.5] | 27.7 | [24.7,30.6] | 22.6 | [20.0,25.2] | 8.9 | [7.1,10.7] |
| Tripura | 19.9 | [19.0,20.7] | 19.8 | [17.6,22.0] | 15.9 | [13.8,17.9] | 5.1 | [4.1,6.2] |
| Meghalaya | 15.6 | [14.6,16.6] | 14.4 | [12.1,16.8] | 17.6 | [14.1,21.1] | 10.5 | [7.3,13.7] |
| Assam | 17.0 | [16.5,17.4] | 20.0 | [18.7,21.2] | 17.6 | [16.4,18.9] | 7.4 | [6.6,8.3] |
| West Bengal | 20.9 | [20.3,21.5] | 16.4 | [15.3,17.5] | 14.9 | [13.9,16.0] | 4.6 | [4.0,5.2] |
| Jharkhand | 14.8 | [14.3,15.4] | 24.5 | [22.0,27.0] | 12.1 | [11.1,13.1] | 4.8 | [4.2,5.3] |
| Odisha | 15.8 | [15.4,16.2] | 22.6 | [21.2,24.1] | 17.4 | [16.5,18.3] | 5.4 | [4.9,5.9] |
| Chhattisgarh | 11.9 | [11.5,12.4] | 24.7 | [22.4,27.1] | 16.7 | [15.4,18.1] | 7.6 | [6.7,8.5] |
| Madhya Pradesh | 12.3 | [12.0,12.7] | 16.6 | [15.7,17.6] | 13.7 | [12.9,14.5] | 5.6 | [5.1,6.1] |
| Gujarat | 17.2 | [16.8,17.6] | 16.7 | [15.8,17.7] | 15.6 | [14.7,16.6] | 5.3 | [4.8,5.8] |
| Daman and Diu and Dadra and Nagar Haveli | 19.1 | [17.5,20.6] | 16.9 | [13.2,20.7] | 19.2 | [15.2,23.1] | 5.8 | [3.9,7.7] |
| Ladakh | 13.2 | [12.8,13.6] | 24.9 | [23.5,26.3] | 23.3 | [22.0,24.7] | 8.9 | [8.1,9.7] |
| Maharashtra | 20.5 | [20.0,21.1] | 33.4 | [31.8,35.0] | 31.5 | [29.9,33.0] | 8.6 | [7.7,9.4] |
| Andhra Pradesh | 15.3 | [14.8,15.7] | 29.3 | [27.7,30.9] | 27.2 | [25.7,28.6] | 8.3 | [7.6,9.1] |
| Karnataka | 21.8 | [20.4,23.1] | 36.5 | [33.1,39.8] | 34.9 | [31.7,38.1] | 10.2 | [8.1,12.2] |
| Goa | 20.8 | [18.4,23.2] | 38.7 | [33.7,43.7] | 32.1 | [27.8,36.4] | 7.7 | [5.8,9.6] |
| Lakshadweep | 23.2 | [22.6,23.7] | 49.1 | [47.8,50.4] | 41.2 | [39.9,42.6] | 11.7 | [10.9,12.6] |
| Kerala | 22.3 | [21.5,23.0] | 44.3 | [42.4,46.2] | 31.2 | [30.1,32.2] | 8.2 | [7.6,8.7] |
| Tamil Nadu | 21.0 | [19.4,22.6] | 44.7 | [40.8,48.6] | 39.4 | [36.4,42.5] | 11.9 | [9.7,14.0] |
| Puducherry | 18.1 | [16.4,19.9] | 37.6 | [34.4,40.7] | 35.2 | [32.2,38.2] | 9.6 | [7.4,11.8] |
| Andaman & Nicobar Islands | 16.7 | [16.3,17.1] | 33.5 | [32.2,34.8] | 31.0 | [29.8,32.2] | 10.9 | [10.0,11.8] |
| Telangana | 12.8 | [9.8,15.8] | 54.4 | [40.3,68.4] | 19.0 | [12.3,25.6] | 9.6 | [5.6,13.6] |

**Supplementary Table S3: Adjusted concentration indices for Diabetes prevalence and ATC among those with diabetes, across states in India, 2019-21**

| **States** | **Prevalence** | **95% CI** | **Awareness** | **95% CI** | **Treatment** | **95% CI** | **Control** | **95% CI** |
| --- | --- | --- | --- | --- | --- | --- | --- | --- |
| Ladakh | -0.020 | [-0.029,0.016] | -0.038 | [-0.080,0.005] | 0.001 | [-0.028,0.032] | -0.010 | [-0.032,0.013] |
| Chandigarh | 0.001 | [-0.014,0.020] | 0.037 | [-0.002,0.077] | 0.036 | [-0.002,0.074] | 0.003 | [-0.015,0.021] |
| Lakshadweep | 0.006 | [-0.008,0.010] | -0.014 | [-0.053,0.025] | -0.016 | [-0.054,0.022] | -0.008 | [-0.030,0.013] |
| Jammu & Kashmir | 0.007 | [0.005,0.010] | 0.010 | [-0.004,0.023] | 0.023 | [0.011,0.035] | 0.000 | [-0.009,0.009] |
| Rajasthan | 0.008 | [0.006,0.020] | 0.092 | [0.083,0.101] | 0.057 | [0.050,0.065] | 0.022 | [0.017,0.027] |
| Goa | 0.010 | [-0.001,0.015] | 0.035 | [0.010,0.061] | 0.042 | [0.017,0.068] | 0.012 | [-0.004,0.028] |
| Delhi | 0.011 | [0.007,0.015] | 0.034 | [0.020,0.047] | 0.025 | [0.012,0.039] | 0.001 | [-0.008,0.010] |
| Madhya Pradesh | 0.013 | [0.011,0.018] | 0.093 | [0.087,0.100] | 0.070 | [0.064,0.077] | 0.020 | [0.015,0.010] |
| Kerala | 0.013 | [0.008,0.017] | 0.041 | [0.028,0.052] | 0.052 | [0.040,0.063] | 0.007 | [0.000,0.010] |
| Punjab | 0.014 | [0.011,0.017] | 0.041 | [0.032,0.051] | 0.038 | [0.029,0.047] | 0.003 | [-0.002,0.010] |
| Haryana | 0.014 | [0.011,0.024] | 0.054 | [0.044,0.065] | 0.043 | [0.034,0.052] | 0.012 | [0.007,0.010] |
| Daman and Diu and Dadra and Nagar Haveli | 0.015 | [0.005,0.019] | 0.094 | [0.073,0.116] | 0.078 | [0.055,0.100] | 0.011 | [-0.003,0.010] |
| Meghalaya | 0.015 | [0.011,0.019] | 0.067 | [0.055,0.079] | 0.042 | [0.029,0.055] | 0.013 | [0.002,0.010] |
| Arunachal Pradesh | 0.016 | [0.013,0.020] | 0.078 | [0.067,0.090] | 0.037 | [0.029,0.046] | 0.009 | [0.003,0.010] |
| Uttar Pradesh | 0.019 | [0.017,0.023] | 0.037 | [0.031,0.043] | 0.044 | [0.039,0.048] | 0.003 | [-0.001,0.010] |
| West Bengal | 0.019 | [0.016,0.023] | 0.042 | [0.034,0.049] | 0.039 | [0.031,0.046] | 0.009 | [0.004,0.010] |
| Gujarat | 0.021 | [0.018,0.027] | 0.069 | [0.063,0.076] | 0.069 | [0.063,0.075] | 0.020 | [0.016,0.010] |
| Mizoram | 0.021 | [0.016,0.027] | 0.109 | [0.093,0.127] | 0.101 | [0.085,0.117] | 0.039 | [0.028,0.010] |
| Chhattisgarh | 0.024 | [0.022,0.030] | 0.102 | [0.092,0.113] | 0.074 | [0.065,0.083] | 0.025 | [0.019,0.010] |
| Nagaland | 0.025 | [0.021,0.034] | 0.107 | [0.092,0.121] | 0.054 | [0.042,0.066] | 0.013 | [0.007,0.010] |
| Puducherry | 0.026 | [0.017,0.035] | 0.056 | [0.035,0.076] | 0.071 | [0.051,0.091] | -0.001 | [-0.014,0.010] |
| Tripura | 0.029 | [0.022,0.032] | 0.085 | [0.072,0.099] | 0.078 | [0.066,0.091] | 0.019 | [0.012,0.010] |
| Jharkhand | 0.029 | [0.026,0.032] | 0.100 | [0.090,0.110] | 0.074 | [0.066,0.081] | 0.020 | [0.015,0.010] |
| Maharashtra | 0.030 | [0.028,0.039] | 0.083 | [0.074,0.091] | 0.079 | [0.071,0.087] | 0.020 | [0.015,0.010] |
| Sikkim | 0.030 | [0.022,0.033] | 0.050 | [0.023,0.078] | 0.048 | [0.022,0.075] | -0.005 | [-0.022,0.010] |
| Assam | 0.031 | [0.028,0.034] | 0.073 | [0.066,0.081] | 0.037 | [0.030,0.044] | -0.003 | [-0.008,0.010] |
| Bihar | 0.031 | [0.029,0.037] | 0.075 | [0.068,0.082] | 0.047 | [0.041,0.054] | 0.005 | [0.001,0.010] |
| Manipur | 0.031 | [0.026,0.036] | 0.096 | [0.079,0.113] | 0.082 | [0.067,0.097] | 0.019 | [0.009,0.010] |
| Karnataka | 0.033 | [0.030,0.039] | 0.063 | [0.054,0.072] | 0.068 | [0.059,0.076] | 0.011 | [0.005,0.010] |
| Himachal Pradesh | 0.035 | [0.030,0.045] | 0.067 | [0.052,0.082] | 0.070 | [0.057,0.083] | 0.018 | [0.010,0.010] |
| Andaman & Nicobar Islands | 0.035 | [0.025,0.039] | 0.083 | [0.055,0.112] | 0.085 | [0.057,0.114] | 0.004 | [-0.013,0.010] |
| Tamil Nadu | 0.036 | [0.032,0.040] | 0.067 | [0.059,0.076] | 0.074 | [0.066,0.081] | 0.010 | [0.005,0.010] |
| Uttarakhand | 0.036 | [0.032,0.043] | 0.119 | [0.103,0.134] | 0.106 | [0.092,0.120] | 0.026 | [0.016,0.010] |
| Andhra Pradesh | 0.038 | [0.033,0.042] | 0.078 | [0.065,0.091] | 0.074 | [0.061,0.087] | 0.007 | [-0.001,0.010] |
| Telangana | 0.039 | [0.036,0.046] | 0.111 | [0.102,0.121] | 0.117 | [0.108,0.126] | 0.028 | [0.022,0.010] |
| Odisha | 0.043 | [0.040,0.046] | 0.109 | [0.100,0.117] | 0.095 | [0.088,0.103] | 0.020 | [0.016,0.010] |

**Supplementary Table S4: Averaged marginal effects for Diabetes Prevalence and ATC among those with diabetes in India, 2019-21**

|  | **Prevalence** | | | | **Awareness** | | | | **Treatment** | | | | **Control** | | | |
| --- | --- | --- | --- | --- | --- | --- | --- | --- | --- | --- | --- | --- | --- | --- | --- | --- |
| **Characteristics** | **Margins** | **p value** | **95% CI** | | **Margins** | **p value** | **95% CI** | | **Margins** | **p value** | **95% CI** | | **Margins** | **p value** | **95% CI** | |
| **Wealth Index** |  |  |  |  |  |  |  |  |  |  |  |  |  |  |  |  |
| Poorest | ref |  |  |  | ref |  |  |  | ref |  |  |  | ref |  |  |  |
| Poorer | 0.011 | 0.000 | 0.008 | 0.013 | 0.035 | 0.000 | 0.027 | 0.042 | 0.019 | 0.000 | 0.013 | 0.025 | 0.005 | 0.045 | 0.000 | 0.010 |
| Middle | 0.023 | 0.000 | 0.021 | 0.026 | 0.063 | 0.000 | 0.055 | 0.071 | 0.044 | 0.000 | 0.038 | 0.050 | 0.008 | 0.004 | 0.003 | 0.013 |
| Richer | 0.039 | 0.000 | 0.036 | 0.043 | 0.092 | 0.000 | 0.083 | 0.101 | 0.072 | 0.000 | 0.065 | 0.079 | 0.012 | 0.000 | 0.006 | 0.017 |
| Richest | 0.057 | 0.000 | 0.053 | 0.061 | 0.124 | 0.000 | 0.113 | 0.134 | 0.105 | 0.000 | 0.097 | 0.114 | 0.023 | 0.000 | 0.016 | 0.030 |
| **Age** |  |  |  |  |  |  |  |  |  |  |  |  |  |  |  |  |
| 15-29 | ref |  |  |  | ref |  |  |  | ref |  |  |  | ref |  |  |  |
| 30-44 | 0.066 | 0.000 | 0.064 | 0.068 | 0.017 | 0.000 | 0.008 | 0.025 | 0.011 | 0.003 | 0.004 | 0.018 | -0.037 | 0.000 | -0.043 | -0.031 |
| 45-59 | 0.175 | 0.000 | 0.172 | 0.178 | 0.104 | 0.000 | 0.095 | 0.114 | 0.105 | 0.000 | 0.097 | 0.113 | -0.023 | 0.000 | -0.030 | -0.016 |
| 60-74 | 0.249 | 0.000 | 0.245 | 0.253 | 0.163 | 0.000 | 0.152 | 0.173 | 0.163 | 0.000 | 0.153 | 0.172 | -0.003 | 0.433 | -0.010 | 0.004 |
| 75+ | 0.254 | 0.000 | 0.247 | 0.261 | 0.155 | 0.000 | 0.141 | 0.169 | 0.152 | 0.000 | 0.139 | 0.165 | 0.003 | 0.487 | -0.006 | 0.013 |
| **Education Level** |  |  |  |  |  |  |  |  |  |  |  |  |  |  |  |  |
| No Education | ref |  |  |  | ref |  |  |  | ref |  |  |  | ref |  |  |  |
| Primary | 0.014 | 0.000 | 0.011 | 0.016 | 0.030 | 0.000 | 0.024 | 0.036 | 0.019 | 0.000 | 0.014 | 0.024 | 0.007 | 0.001 | 0.003 | 0.011 |
| Secondary | 0.014 | 0.000 | 0.011 | 0.016 | 0.045 | 0.000 | 0.039 | 0.051 | 0.033 | 0.000 | 0.028 | 0.038 | 0.013 | 0.000 | 0.009 | 0.017 |
| Higher | 0.006 | 0.001 | 0.002 | 0.010 | 0.054 | 0.000 | 0.044 | 0.063 | 0.023 | 0.000 | 0.015 | 0.030 | 0.009 | 0.003 | 0.003 | 0.014 |
| **Residential Status** |  |  |  |  |  |  |  |  |  |  |  |  |  |  |  |  |
| Urban | ref |  |  |  | ref |  |  |  | ref |  |  |  | ref |  |  |  |
| Rural | -0.008 | 0.000 | -0.010 | -0.005 | -0.013 | 0.001 | -0.021 | -0.006 | -0.016 | 0.000 | -0.021 | -0.011 | -0.004 | 0.054 | -0.008 | 0.000 |
| **Caste** |  |  |  |  |  |  |  |  |  |  |  |  |  |  |  |  |
| Schedule Caste | ref |  |  |  | ref |  |  |  | ref |  |  |  | ref |  |  |  |
| Schedule Tribe | -0.006 | 0.001 | -0.010 | -0.003 | -0.038 | 0.000 | -0.050 | -0.027 | -0.025 | 0.000 | -0.033 | -0.016 | -0.009 | 0.008 | -0.016 | -0.002 |
| OBC | 0.001 | 0.308 | -0.001 | 0.004 | -0.007 | 0.043 | -0.014 | 0.000 | -0.003 | 0.297 | -0.008 | 0.003 | -0.003 | 0.191 | -0.008 | 0.002 |
| Other | 0.011 | 0.000 | 0.008 | 0.014 | 0.010 | 0.013 | 0.002 | 0.018 | 0.010 | 0.002 | 0.003 | 0.016 | 0.001 | 0.644 | -0.004 | 0.006 |
| **Religion** |  |  |  |  |  |  |  |  |  |  |  |  |  |  |  |  |
| Hindus | ref |  |  |  | ref |  |  |  | ref |  |  |  | ref |  |  |  |
| Muslims | 0.010 | 0.000 | 0.006 | 0.013 | 0.026 | 0.000 | 0.017 | 0.036 | 0.023 | 0.000 | 0.015 | 0.030 | 0.002 | 0.526 | -0.004 | 0.007 |
| Christians | 0.012 | 0.000 | 0.006 | 0.018 | 0.032 | 0.000 | 0.016 | 0.048 | 0.019 | 0.001 | 0.008 | 0.030 | 0.009 | 0.042 | 0.000 | 0.017 |
| Others | -0.003 | 0.341 | -0.009 | 0.003 | 0.007 | 0.329 | -0.007 | 0.022 | 0.000 | 0.952 | -0.011 | 0.012 | -0.005 | 0.338 | -0.015 | 0.005 |
| **Marital Status** |  |  |  |  |  |  |  |  |  |  |  |  |  |  |  |  |
| Unmarried | ref |  |  |  | ref |  |  |  | ref |  |  |  | ref |  |  |  |
| Married | 0.043 | 0.000 | 0.040 | 0.046 | 0.043 | 0.000 | 0.033 | 0.053 | 0.005 | 0.273 | -0.004 | 0.014 | -0.010 | 0.001 | -0.017 | -0.004 |
| Other | 0.051 | 0.000 | 0.047 | 0.055 | 0.039 | 0.000 | 0.027 | 0.051 | 0.005 | 0.352 | -0.005 | 0.015 | -0.008 | 0.044 | -0.016 | 0.000 |
| **Alcohol Usage** |  |  |  |  |  |  |  |  |  |  |  |  |  |  |  |  |
| Not Drinking | ref |  |  |  | ref |  |  |  | ref |  |  |  | ref |  |  |  |
| Drinking | -0.004 | 0.001 | -0.007 | -0.002 | -0.016 | 0.000 | -0.023 | -0.009 | -0.020 | 0.000 | -0.025 | -0.014 | -0.008 | 0.002 | -0.013 | -0.003 |
| **Tobacco Use** |  |  |  |  |  |  |  |  |  |  |  |  |  |  |  |  |
| Not Using | ref |  |  |  | ref |  |  |  | ref |  |  |  | ref |  |  |  |
| Using | -0.005 | 0.000 | -0.007 | -0.003 | -0.026 | 0.000 | -0.032 | -0.021 | -0.022 | 0.000 | -0.026 | -0.017 | -0.006 | 0.001 | -0.010 | -0.002 |
| **Sex** |  |  |  |  |  |  |  |  |  |  |  |  |  |  |  |  |
| Male | ref |  |  |  | ref |  |  |  | ref |  |  |  | ref |  |  |  |
| Female | -0.018 | 0.000 | -0.020 | -0.016 | 0.025 | 0.000 | 0.020 | 0.029 | 0.010 | 0.000 | 0.006 | 0.014 | 0.008 | 0.000 | 0.004 | 0.011 |
| **Household Size** |  |  |  |  |  |  |  |  |  |  |  |  |  |  |  |  |
| Less Than 3 | ref |  |  |  | ref |  |  |  | ref |  |  |  | ref |  |  |  |
| 4-6 | -0.013 | 0.000 | -0.015 | -0.011 | -0.011 | 0.000 | -0.016 | -0.006 | -0.009 | 0.000 | -0.014 | -0.005 | -0.001 | 0.414 | -0.005 | 0.002 |
| 7+ | -0.012 | 0.000 | -0.015 | -0.009 | -0.008 | 0.034 | -0.016 | -0.001 | -0.010 | 0.003 | -0.016 | -0.003 | 0.000 | 0.944 | -0.006 | 0.005 |
